# Quantum Molecular Resonance Radiofrequency Disc Decompression and Percutaneous Microdiscectomy for Lumbar Radiculopathy

**DOI:** 10.3390/jcm13010234

**Published:** 2023-12-30

**Authors:** Ángeles Canós-Verdecho, Ruth Robledo, Rosa M. Izquierdo, Ara Bermejo, Elisa Gallach, David Abejón, María Pilar Argente-Navarro, Isabel Peraita-Costa, María Morales-Suárez-Varela

**Affiliations:** 1 Multidisciplinary Pain Management Unit, Hospital Universitari i Politècnic La Fe, Av. de Fernando Abril Martorell, 106, 46026 Valencia, Spainrobledo_rut@gva.es (R.R.);; 2 Anaesthesiology Department, Hospital Universitari i Politècnic La Fe, Av. de Fernando Abril Martorell, 106, 46026 Valencia, Spain; 3 Psychiatry Department, Hospital Universitari i Politècnic La Fe, Av. de Fernando Abril Martorell, 106, 46026 Valencia, Spain; 4 Multidisciplinary Pain Management Unit, Hospital Universitario Quirónsalud, Calle Diego de Velázquez, 1, 28223 Pozuelo de Alarcón, Spain; 5 Surgical Specialties Department, Hospital Universitari i Politècnic La Fe, Av. de Fernando Abril Martorell, 106, 46026 Valencia, Spain; 6 Research Group in Social and Nutritional Epidemiology, Pharmacoepidemiology and Public Health, Department of Preventive Medicine and Public Health, Food Sciences, Toxicology and Forensic Medicine, Faculty of Pharmacy, Universitat de València, Av. Vicent Andrés Estellés s/n, 46100 Burjassot, Spain; 7 CIBER Epidemiology and Public Health (CIBERESP), Carlos III Health Institute (ISCIII), Av. Monforte de Lemos, 3-5, Pabellón 11, Planta 0, 28029 Madrid, Spain

**Keywords:** analgesia, back pain, pain management, treatment outcome

## Abstract

Within the practice of pain management, one of the most commonly encountered events is low back pain. Lumbar radiculopathy (LR) is a pain syndrome caused by the compression or irritation of the nerve roots in the lower back due to lumbar disc herniation, vertebra degeneration, or foramen narrowing. Symptoms of LR include low back pain that propagates toward the legs, numbness, weakness, and loss of reflexes. The aim of this study is to assess the long-term effectiveness of quantum molecular resonance disc decompression and its combination with a percutaneous microdiscectomy using Grasper© forceps (QMRG) in patients with persistent lumbar radiculopathy (LR) in relation to patient physical stress status. The main outcome measures of this prospective observational study were DN4, NRS, ODI, SF12, PGI, CGI, and MOS Sleep Scale. An improvement 12 months post-intervention was observed in patients without physical stress, presenting better overall results. The mean change was over the minimal clinically important difference in 64.3% of outcome measures studied for the whole sample. QMRG appears to be an effective treatment option for LR, but a reduction in physical stress is needed to ensure long-term effectiveness.

## 1. Introduction

Lumbar radiculopathy (LR) is a pain disorder that affects the lower spine and is caused by the impingement and/or inflammation of the spinal nerve roots [1]. LR is one of the most common causes of low back pain, possibly, and it is estimated that in industrialized countries, eight out of ten people will be affected at some point during their life [2]. The most common underlying cause of LR is lumbar disc bulging or herniation with nerve compression [1]. This back and/or leg pain is normally caused by the herniation of the nucleus pulposus due to inflammation and/or compression of nerve roots as it passes adjacent to the herniated disc [3]. The compression may occur within the thecal sac, within the lateral recess as the nerve root exits the thecal sac, as the nerve root traverses the neural foramina, or even after the nerve root has exited the foramina. LR can also be caused by the degeneration of the spinal vertebra, the narrowing of the vertebral foramen, facet or ligamentous hypertrophy, spondylolisthesis, nerve root injuries, scar tissue from previous spinal surgery, diabetes, or even neoplastic and infectious processes [1]. In contained disc herniations, the nerve roots become irritated by the nucleus material within the posterior annulus, and this is what is believed to cause pain [3]. Symptoms include electric, burning, or sharp low back pain or irritation that radiates into the buttock and down the legs following a dermatomal pattern. Other symptoms may include but are not limited to numbness, tingling, weakness, and loss of specific reflexes [4,5].

Data available from epidemiological studies on the incidence of low back pain presents estimates varying from 5% to more than 30%, with an estimated lifetime prevalence between 60% and 90% [1]. Most cases are self-limiting, and the pain will disappear without the need for intervention [1]. About half of the cases will resolve within 10–15 days, and within six to twelve weeks around 90% of cases are expected to have remitted [1]. For the cases of persistent LR resistant to more traditional or conservative treatments for which surgery is not indicated, minimally invasive decompressive techniques of the disc that are aimed at relieving pressure within the disc tissue appear to be an effective alternative with a low rate of complications. 

Quantum Molecular Resonance^®^ (QMR) is an innovative and unique technology, originally developed and patented by Telea Electronic Engineering Srl (Vicenza, Italy), and is now used in various clinical procedures, including tonsillectomy, dura mate cutting in neurosurgery, insertion of bone anchored hearing aids and epiduroscopy [6,7,8,9,10,11,12]. What makes QMR technology unique and innovative is the ability to cut and/or vaporize biological tissue through the use of different frequencies transmitted simultaneously instead of using heat, which can cause irreparable thermal damage and result in necrosis. QMR energy is generated by means of alternate currents and high-frequency electron waves and characterized at precise and well-defined 8-, 12-, and 16-MHz waves with decreasing amplitudes [6]. This process creates energy quanta that break the molecular bonds without increasing the kinetic energy of the molecules and, therefore, not raising the temperature. Electrosurgical devices that use QMR allow surgeons to maintain the biological structure of the tissue as the functionality is provided at a temperature below 50 °C. QMR can be used for percutaneous disc decompression as it causes the hernia or protrusion to retract to its natural position, reducing compression and inflammation of the adjacent nerve roots [13,14,15]. However, contrary to other techniques used for percutaneous disc decompression, such as laser or radiofrequency, evidence on the effectiveness of QMR is scarce and requires further research.

Therefore, in this prospective observational study, the long-term effectiveness of QMR disc decompression and its combination with a microdiscectomy using Grasper^®^ forceps (QMRG) was assessed in patients with resistant LR secondary to contained lumbar disc hernia or lumbar disc protrusion with more than 6 months of evolution in relation to their physical stress status.

## 2. Patients and Methods

### 2.1. Study Design

This epidemiological observational longitudinal before-after intervention study evaluated patients at the Multidisciplinary Pain Management Unit of the La Fe University and Polytechnic Hospital in Valencia between July 2021 and December 2022. Approval was obtained from the “Comité de Ética de la Investigación con medicamentos del Instituto de Investigación Sanitaria La Fe” (Registration number 2021-341-1). The study adheres to the principles for research in humans outlined in the Declaration of Helsinki. The protocol was explained to all patients, and written informed consent was obtained at the beginning of the study.

### 2.2. Participants

The participants of this study were over 18 years of age, had LR secondary to an L4-L5/L5-S1 contained hydrated lumbar disc hernia or lumbar disc protrusion with symptoms lasting more than 6 months and were resistant to treatment with analgesia, physical therapy, and/or epidural block. Participants had undergone QMRG and completed a 12-month follow-up protocol within the last two years in the Multidisciplinary Pain Management Unit of the La Fe University and Polytechnic Hospital in Valencia (MPMU). 

**Figure 1:**
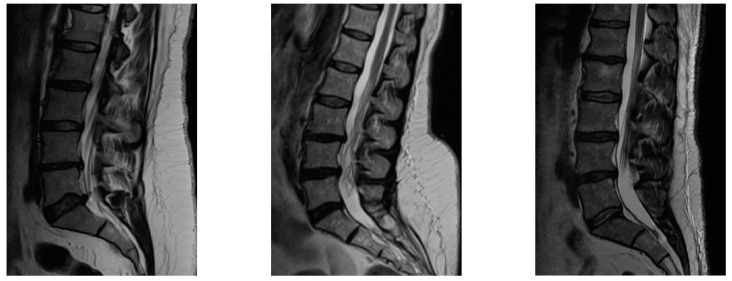
Sample MRI imaging of treated hernias/protrusions.

Initial diagnosis for LR was based on clinical presentation, magnetic resonance imaging (MRI) and electromyography (EMG) results. A sample of MRI images of the treated hernias/protrusions can be seen in Figure 1. Only in patients that reported no improvement after treatment with QMRG was further MRI testing performed.

The required sample size was calculated using G*Power (version 3.1.9.7) software (available freely from Heinrich Heine Universität at https://www.psychologie.hhu.de/arbeitsgruppen/allgemeine-psychologie-und-arbeitspsychologie/gpower.html, accessed on 15 November 2023). The primary outcome was the difference between the two groups with respect to the outcome measure scores, assessed using different questionnaires and scales. It was estimated that a sample size of 21 patients per group would achieve a power of 80% to detect a large effect size (Cohen’s d) of 0.8. The statistical test used was the two-sided unpaired *t*-test, and the type I error was set at 0.05. 

After the establishment of the minimum required sample size, the hospital’s electronic records were searched for patients that met all inclusion criteria and could potentially be included in this study. This search yielded a total of 49 candidate patients. Potential participants were contacted and received detailed information about the study. Along with the previously mentioned diagnostic and treatment criteria, the following requisites were established for inclusion in the study:

- Patient confirmation of their understanding regarding the study protocol.
- Written and signed patient informed consent form.
- Patient ability and willingness to follow study protocol and requirements.

Of the 49 patients identified as potentially eligible for inclusion, three were uncontactable, and two did not give informed consent. Therefore, leaving a sample of 44 patients who were considered to meet all the inclusion criteria.

After all relevant medical data was collected from the electronic records, the data was first reviewed to ensure that none of the previously excluded criteria were present at the time of intervention, in the six months leading up to the intervention, or during the 12-month follow-up period. The exclusion criteria specific to the moment of the intervention were:

- Prior treatment by discectomy or percutaneous microdiscectomy, with or without a disc prosthesis.
- Systemic infection.
- Active skin infection in the access area of the technique.

The following were the exclusion criteria in the six months preceding the intervention and/or during the 12-month follow-up period:

- History or active treatment of cancer.
- Documented history of substance abuse or dependence.
- Immunosuppression.
- Severe neurological or psychiatric pathology.
- Anticoagulant therapy.
- Pregnancy.
- Participation in other clinical research with an active treatment group.

One patient met any of the exclusion criteria and, therefore, was excluded, leaving a final sample of 43 patients, with full data at baseline, 1-month, 6-months, and 12-months. Patients then underwent treatment with QMRG and completed a 12-month follow-up with the Multidisciplinary Pain Management Unit of the La Fe University and Polytechnic Hospital in Valencia (MPMU). These patients constituted the single cohort that was evaluated in relation to their physical stress status in this study.

As with other musculoskeletal system disorders, LR can have a complex multi-factorial root cause determined by different biomechanical, hereditary, developmental, hormonal, vascular, autoimmune, and adverse environmental conditions that trigger the occurrence of the root cause and exacerbate the associated pain [16]. One of the principal factors that may induce LR is physical stress, normally of an occupational origin. Physical stress has been defined as lifting/carrying heavy items or cumulative load to the spine, pushing/pulling loads, repeated tasks with the torso bent/twisted, fixed, or awkward body postures, standing/walking, sitting, kneeling/squatting, whole-body and local vibration, the combination of different mechanical exposures, and poor working conditions and/or microclimate [17,18,19].

### 2.3. Intervention and Follow-Up

**Figure 2:**
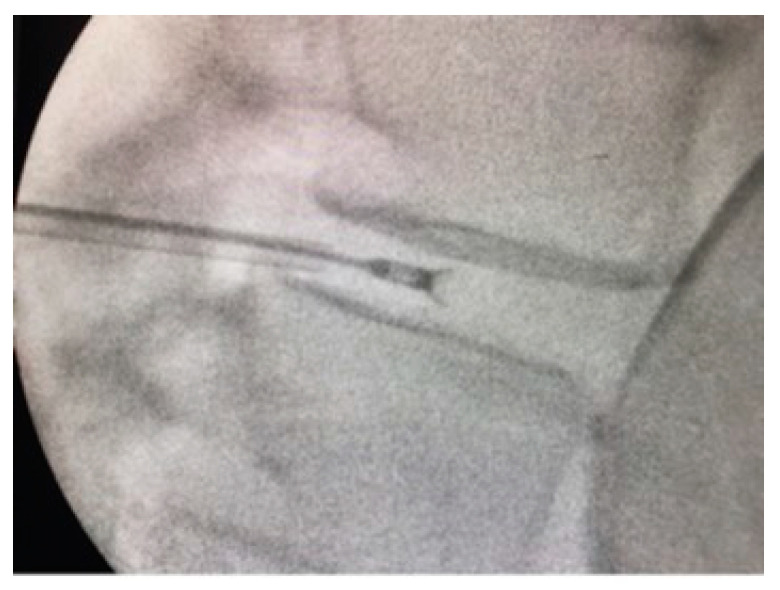
Fluoroscopic image taken during intervention.

The device, intervention, and follow-up protocol have been extensively described in a previous publication. For full details, see Canós-Verdecho et al., 2022 [20]. A fluoroscopic image taken during one of the interventions is shown in Figure 2. Intervention dates were from 8 July 2021 to 9 December 2021, and the final follow-up was completed in December 2022.

### 2.4. Outcome Parameters

The intervention protocol of the MPMU requires that the patient’s evolution be evaluated using several different questionnaires and scales (NRS: Numeric Rating Scale; ODI: Oswestry Disability Index; SF-12: Short Form 12 Health Survey; PGI: Patient Global Impression of Improvement; CGI: Clinical Global Impression of Improvement; and MOS: Medical Outcomes Study Sleep Scale) before the day of intervention and at 1-month, 3-months, 6-months, and 12-months post-intervention. For the MOS, all its constituent components (SLPD4: disturbances, SLPSNR1: snoring, SLPSOB1: shortness of breath or headache, SLPA2: adequacy, SLPS3: somnolence, SLP6: problems index I, and SLP9: problems index II) were evaluated. Demographic data such as age, sex, marital status, education, and employment were recorded during the baseline visit, along with information on pain location and physical stress. The Douleur Neuropathique en 4 Questions (DN4) questionnaire is also administered at the same time.

The minimal difference in outcomes that was considered significant was the minimal clinically important difference (MCID) established for each outcome measure. The MCID is the smallest change considered important or notable by the patient. It is a patient-centered concept that captures the magnitude of change, which can be positive or negative, and the value patients place on this change [21,22,23,24]. MCID values vary depending on the patient, clinical context, and estimation method [25]; however, some outcome measures do have previously established and recognized MCIDs. 

For outcome measures of chronic pain such as DN4 or NRS, a two-point (ten-point scale) or 30% improvement is considered the MCID [26]. The MCID for the ODI used in previous studies ranges from 4 to 15 points [27,28,29,30]. In this study, the ODI MCID has been set at 10 points as a compromise. When it comes to the SF-12 mental and physical component scores, >3.77 in MCS and >3.29 in PCS (normalized 100-point scale) have been established as the MCIDs in patients suffering from chronic lower back pain [31]. Given its sensitivity and strong correlation with patient satisfaction [32], a change of 1 in PGI is generally accepted as the MCID [33,34,35]. A previous study has established an MCID threshold for sleep-related outcomes measured using the MOS Sleep Scale scores (100-point scale) at ≥6.41 [36].

Data was collected prospectively and recorded by specialists at the MPMU. Another physician evaluated all measurements (baseline, after 1 month, after 3 months, after 6 months, and after 12 months). Additionally, any adverse effects were recorded in the patient’s medical history if they occurred.

### 2.5. Statistical Analysis

IBM SPSS version 27.0 (IBM Corp., Armonk, NY, USA) was used for the statistical analysis. A descriptive analysis was performed by calculating the frequency and percentage. For categorical variables, proportions were compared using either the chi-square test for contingency tables or Fisher’s exact test (expected frequencies > 5). 

The normality of the distribution of continuous variables was analyzed using the Shapiro–Wilk test. Normally distributed data were compared using an independent sample *t*-test and presented as mean ± standard deviation and range (minimum and maximum). The Mann–Whitney U-tests were used for nonparametric data, which were presented as median and interquartile range. Results were considered statistically significant when *p* < 0.05. The Freidman and Wilcoxon tests were used to evaluate the significance of the variations between repetitive measurements. In order to avoid possible type I errors, a Bonferroni correction was used, and values *p* < 0.017 were considered statistically significant. 

The trend of the score obtained in each of the assessments carried out over the follow-up time (baseline, 1-month, 3-months, 6-months, and 12-months) was assessed using a repeated-measures one-factor ANOVA test and the mean variations throughout the follow-up period were graphically portrayed via line graphs. Change at the end of follow-up, 12 months, was calculated for each of the outcomes (NRS, ODI, PGI, CGI, SF12 Physical, SF12 Mental, SF12 Total, SLPD4, SLPSNR1, SLPSOB1, SLPA2, SLPS3, SLP6, and SLP9) by the difference in score at 12 months and the baseline assessment. The frequency and percentage of patients exceeding the clinically relevant percentage change for each of the parameter’s studies was then calculated. 

## 3. Results

Forty-three patients met the established inclusion/exclusion criteria. Twenty-one patients (48.8%) were under habitual physical stress, and twenty-two (51.2%) were not. At the time of the intervention, patients presented with neuropathic pain and were receiving pharmacological treatment for it with either anticonvulsants (pregabalin or gabapentin) or antidepressants (duloxetine), which were gradually withdrawn shortly after intervention in most cases and therefore not expected to have an effect on the long-term outcomes studied. General personal baseline characteristics of the sample are shown in Table 1, and no differences were reported among the two groups.

**Figure 3:**
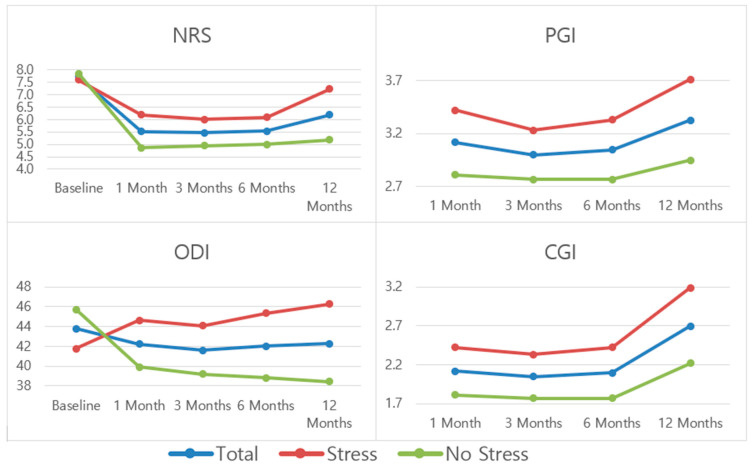
Mean patient scores for NRS, ODI, PGI, and CGI.

**Figure 4:**
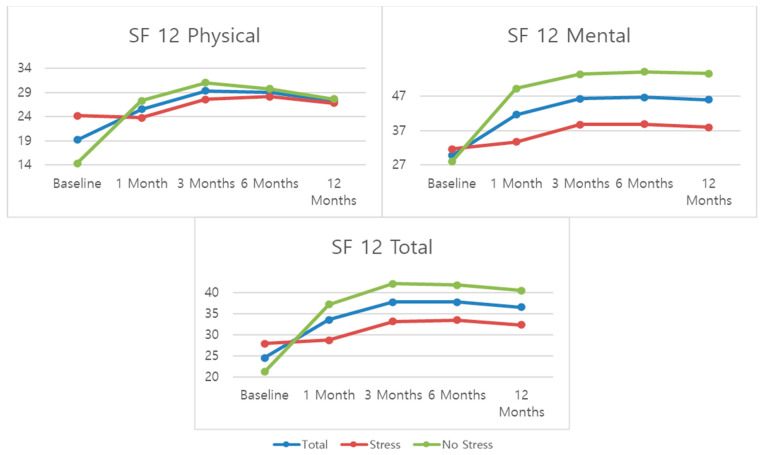
Mean patient scores for SF12.

Table 2 and Figure 3 show the scores for NRS, ODI, PGI, and CGI outcomes at the established follow-up time periods, while Table 3 and Figure 4 present scores at the established follow-up time periods for SF-12. For the scoring trend throughout the follow-up period for the entire sample, statistically significant differences were observed for NRS and all SF-12 outcomes. If the trends among the stress groups are compared, statistically significant differences were observed for all pain and function-related outcome measures except for SF-12 Physical.

For pain (NRS), the biggest improvement is seen at the 1-month mark, followed by a stabilization and slight worsening at the 12-month mark. For disability (ODI), the trends are opposite depending on the stress group. The “stress” group shows a trend for progressive worsening scores, while the “no stress” group shows a slight improvement and stabilization throughout the follow-up period. For both patient and clinician perceived improvement (PGI and CGI), scores are relatively stable at the 1, 3, and 6-month marks and better at the 12-month mark.

For quality of life (SF-12), the biggest improvement is seen at the 1-month mark and from there, the trend is for a stabilization of the scores. Scores for the mental SF-12 subscale are significantly better than those for the physical subscale. When compared by stress groups, the “no stress” group presented significantly better scores for all three SF-12 outcomes than the “stress” group. 

**Figure 5:**
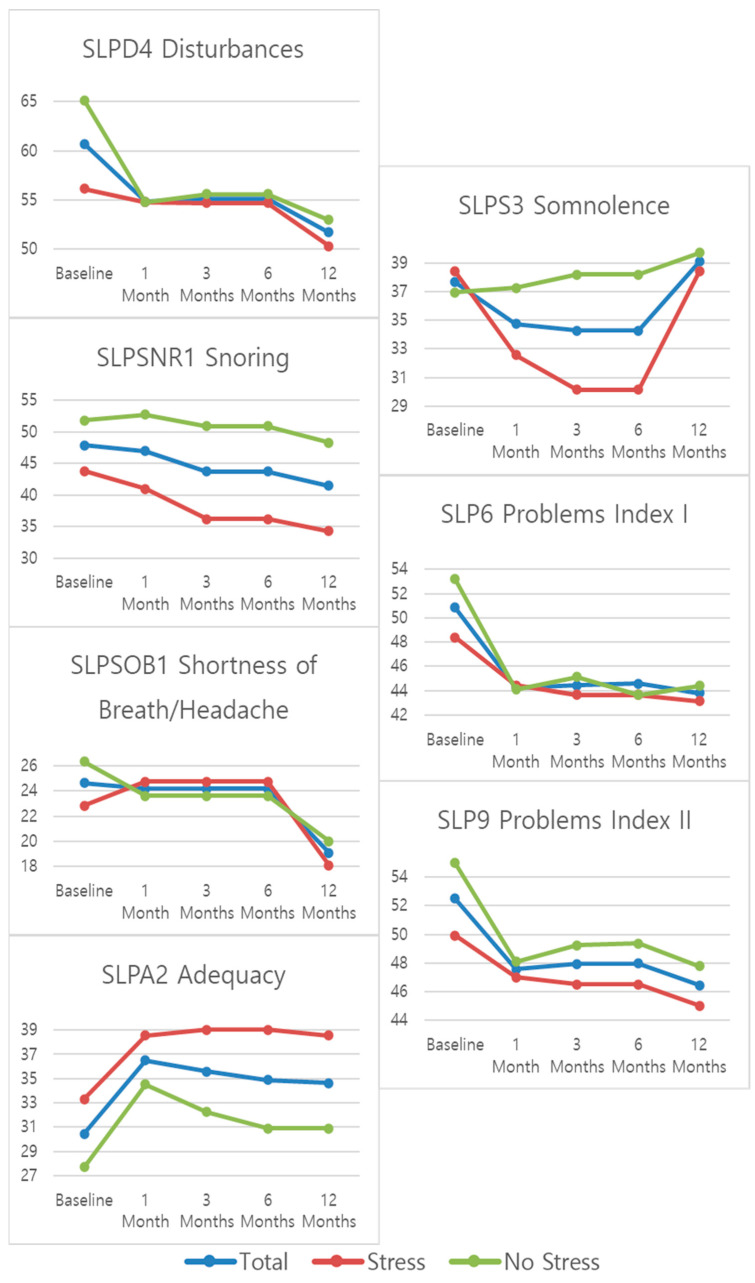
Mean patient scores for MOS Sleep Scale subscales.

Table 4 and Figure 5 show the mean ± standard deviations scores for the MOS Sleep Scale outcomes at the established follow-up time periods. A statistically significant change during the follow-up was observed for all sleep-related outcome measures in at least one of the studied groups except for “sleep adequacy” (SLPA2) and “daytime somnolence” (SLPS3). The trend for all sleep-related outcomes is for improvement at the 12-month mark, with the biggest change observed at the 1-month mark. For “sleep disturbance”, “sleep awakening short of breath or with headache”, and “daytime somnolence”, a significant change can also be observed between the 6- and 12-month marks. For “sleep disturbance” and “sleep awakening short of breath or with headache”, the change is a further improvement in scores, but for “daytime somnolence”, there is a worsening between the 6- and 12-month marks, especially for the “stress” group. When it comes to the sleep problems indexes (SLP6 and SLP9) both present an initial improvement that appears to stabilize over the follow-up period. 

Table 5 shows the differences in the pre- and 12-month post-intervention outcome scores and how these differences relate to the established MCID thresholds for each outcome measure. If considered as a single group, the mean change at 12 months post-intervention is over the MCID in 9 of the 14 outcome measures studied. However, when separated by physical stress status, the “stress” group is over the MCID in 4 outcomes, while the “no stress” group is over the MCID in 10 outcomes. In 3 of the 4 outcomes that the “stress” group meets the MCID thresholds, the improvement in this group is better than that observed in the “no stress” group; the only exception is SF12 Mental. Also, the “stress” group meets the MCID threshold for snoring (SLPSNR1), while the “no stress” group does not.

The results show that 12 months after intervention, the MCID threshold is met by between 14% and 100% of patients, depending on the outcome measure, for the non-sleep-related outcomes and for between 0% and 41.9% of patients for the sleep-related outcome measures. Of the 14 outcome measures studied, in 5 of them, at least 50% of patients met the MCID threshold. When separated by physical stress status, in the “stress” group, the MCID threshold is met by between 14.3% and 100% of patients, depending on the outcome measure, for the non-sleep-related outcomes and for between 0% and 33.3% of patients for the sleep-related outcome measures. In the “no stress” group, the MCID threshold is met by between 4.5% and 100% of patients, depending on the outcome measure, for the non-sleep-related outcomes and for between 0% and 50.0% of patients for the sleep-related outcome measures. Of the 14 outcome measures studied, in 3 of them, at least 50% of patients met the MCID threshold in the “stress” group, and in 5 of them, at least 50% of patients met the MCID threshold in the “no stress” group. All of these outcomes where the MCID threshold was met by at least half of the patients were non-sleep-related.

## 4. Discussion

There is little evidence in the literature to guide LR treatment in patients where non-interventional treatment modalities do not help. When 6 months of conservative therapy, such as exercise, physical therapy, and/or medications, has failed, surgical intervention may be considered. In patients presenting persistent LR, discectomy, either open or microsurgical, is the surgical treatment option most commonly followed and only if further degeneration is present may surgical fusion be necessary [3]. Recently, minimally invasive techniques, such as QMRG, have emerged as effective treatment options in patients with LR, and the goal of these is to bridge the gap between failed conservative treatment and open surgery [3]. Therefore, while open surgery seems to be too invasive and conservative treatment may present unsatisfactory results, evidence from this and another study [18] shows good results for patients with persistent LR secondary to contained hernias who undergo treatment with QMRG which could open the way for a new therapeutic pathway [20].

This study compared the effectiveness of quantum molecular resonance coablative radiofrequency disc decompression and percutaneous microdiscectomy using Grasper forceps in the treatment of lumbar radiculopathy based on pain, function, and sleep depending on physical stress status. The results of this study demonstrated improved pain, function, and sleep outcomes at 12 months for the whole patient sample; however, there were statistically significant differences in improvement between the two stress groups, with the “no stress” group presenting better outcomes at 12 months post-intervention.

As publications on QMR technology use for spinal disc decompression are almost non-existent it is impossible to compare the results of this study regarding the efficacy of QMRG and its dependence on patient physical stress status. Data on other minimally invasive techniques and their dependence on patient physical stress status that could be relevant for comparison is also missing. This only highlights the need for further research into the potential of QMR techniques as a therapeutic option for the treatment of LR. However, studies on different minimally invasive techniques with a significant follow-up time (over 1 year), which could rationally be expected to include the patient returning to ordinary daily and professional activities, have shown that these types of interventions appear to be safe and effective options for percutaneous lumbar disc decompression [37,38,39,40], but once again, no definitive conclusions can be established at this time.

Achieving a successful outcome in a percutaneous lumbar disc decompression depends in part on a careful patient selection, as specific patient profiles may be more suited for this type of intervention. Factors such as disc herniation severity, location comorbid conditions, and patient compliance with treatment protocol have the ability to influence outcomes [15]. Therefore, a thorough assessment of the patient’s profile is critical before any intervention is considered, and further investigation to help establish an optimal candidate profile for the treatment of LR with QMRG is warranted. This identification would improve the chance of success in achieving positive long-term outcomes and could help guide treatment strategies and protocols.

It is advised that patients with low back pain stay active and continue with their ordinary daily activities when possible, as remaining active is associated with shorter recovery times and a lower incidence of recurrent problems [41]. Aerobic exercise, such as walking, is recommended for most patients with LR [42]. This recommendation to remain as active as possible and participate in structured physical activity during the follow-up to the intervention was also given to the participants of this study and was reiterated in all follow-up visits as part of the protocol followed at the MPMU. 

Possibly related to patient activity levels, in this study, the most significant improvement was observed at the 1-month mark, with improvement then plateauing for the most part. The improvement reported by the patients during the first follow-up visit could be related to their perception of or lack of pain or physical limitations while carrying out these ordinary daily activities and exercise plans. Their ability to participate in and complete certain tasks could heighten the perception of improvement and set a new baseline for the patients. Therefore, it would appear logical that they report a greater improvement immediately after intervention and then reset their expectations which could influence perceived improvement or worsening later on during follow-up. 

It may be necessary to reset realistic expectations and goals in the patient by the management team, as it is important to educate patients on the fact that sporadic or temporary mild pain does not necessarily mean an overall worsening of the condition and that it can actually be beneficial to the patient to experience some short-term pain. For example, physical activity and exercise can be painful for a patient with LR, but it is beneficial to their overall recovery to maintain as normal a functional status as possible.

In a study by Kerr et al. [43] that assessed outcomes of operative and nonoperative treatment for patients with intervertebral disc herniation and symptomatic radiculopathy at 8 years, patients who were working showed the biggest relative improvements compared to those who were work-disabled. This, however, may also be because those employed presented less complicated or severe cases of herniation.

For patients with persistent pain due to LR, a return to work after treatment may expose them to the occupational exposures responsible for the root cause of their LR. The physical demands of the patient’s job when recommending activity for the employed patient with low back symptoms must be considered [41]. Occupational physical stress has been reported to be associated with disc degeneration [44,45], and in previous studies, the specific type of work performed is unknown. Prolonged sitting, twisting/bending, lifting heavy objects, and heavy physical load have been previously associated with disc degeneration [46]. At the L4-L5 level, occupational mechanical lumbar stress has been shown to possibly accelerate disc degeneration [47]. Modifications in work activities are often necessary in patients with LR and should be tailored individually according to the physical requirements of their job, the severity of their condition and their understanding of their condition and limitations [42].

In a non-occupational setting, lifting, especially when performed with straight knees and a bent back, has also been associated with an increased risk of herniated lumbar disc [48]. In general, activities that cause a significant increase in symptoms should be modified, but at the same time, it should be noted that some “popular” actions, such as maintaining lordotic postures or certain sleep postures, have not been proven to be beneficial for the treatment and prevention of low back pain [42]. 

The return to a physically stressful environment, occupational or not, or more vigorous physical activity after effective treatment with QMRG without any occupational adaptations to prevent a recurrence of the lumbar disc herniation or protrusion may be responsible for the negative trend observed for some of the outcome measures between the 6 and 12-months marks. The outcomes where this trend appears are NRS, SF-12 Physical, SLPS3 and ODI only for the “stress” group. In all cases, the worsening of the scores is more pronounced in the “stress” group, which would support this idea. Given this and to ascertain the durability and possible predictors of outcomes, long-term follow-up studies are needed.

### Limitations

The small sample size of the study is one of its main limitations. The QMRG technique is novel, and, therefore, the pool of patients with the required minimum follow-up time is also small. One of the limitations of the available sample size is, for example, that a specific patient profile based on personal characteristics, other than physical stress status, most likely to benefit from treatment with QMRG could not be established. However, it should also be noted that limiting patients’ physical stress for a period as long as twelve months is often not feasible, and the evidence of better clinical outcomes when physical stress is limited would suggest that QMRG may be more effective in a certain population, such as elderly or specific types of workers. This could be a disadvantage when compared to regular discectomy, as outcomes after that type of intervention have been shown to not be dependent on physical duties [49]. 

Another limitation is the possible selection bias derived from the differing clinical judgments of the healthcare professionals involved. Treatment bias and/or placebo effect could be present given that it is not possible to blind treatment to either the health care professionals or patients. Finally, it cannot be ruled out that the general improvement in outcome measures after treatment with QMRG shown in this study might also be related to normal body recovery post-intervention and not directly to the intervention itself. Therefore, while it is understandable that this was an observational before-after intervention study, with ethical considerations, a randomized control trial that compares a group that underwent QMRG with a control group that had discectomy with no QMRG could be an approach that should be considered in future studies. 

## 5. Conclusions

In conclusion, this study demonstrated a general improvement in outcome measures in patients with LR secondary to a contained lumbar disc hernia or lumbar disc protrusion treated with QMRG at 12 months following intervention with those patients without physical stress presenting better overall results. With the results observed in this study, QMRG appears to be an effective alternative treatment option for LR, but the need for lifestyle and occupational adaptations to reduce the physical stress load on patients must also be considered in order to ensure its long-term positive effects in patients and prevent the recurrence of LR.

## Figures and Tables

**Table 1:** Baseline characteristics of the patients.

	Total	Stress	No Stress	*p*-Value *
	N = 43 (100)	N = 21 (48.8)	N = 22 (51.2)	
**AGE (years) at Enrollment**				
Mean ± SD (Range)	51.90 ± 9.73 (31.00–74.00)	52.38 ± 9.38 (35.00–74.00)	51.45 ± 10.25 (31.00–69.00)	0.759
**SEX** *n* (%)				0.069
Male	22 (51.2)	14 (66.7)	8 (36.4)	
Female	21 (48.8)	7 (33.3)	14 (63.6)	
**Pain Location** *n* (%)				0.603
L3-L4	4 (9.3)	3 (14.3)	1 (4.5)	
L3-L4-L5	4 (9.3)	3 (14.3)	1 (4.5)	
L4-L5	26 (60.5)	11 (52.4)	15 (68.2)	
L4-L5-S1	2 (4.7)	1 (4.8)	1 (4.5)	
L5-S1	7 (16.3)	3 (14.3)	4 (18.2)	
**DN4**				
Mean ± SD (Range)	4.37 ± 1.09 (0–5)	4.19 ± 1.32 (0–5)	4.54 ± 0.80 (0–5)	0.292
**MARITAL STATUS** *n* (%)				0.080
Single	8 (18.6)	4 (19.0)	4 (18.2)	
Married	29 (67.4)	11 (52.4)	18 (81.8)	
Divorced	6 (14.0)	6 (28.6)	0 (0.0)	
**EDUCATION** *n* (%)				0.095
Primary	9 (20.9)	4 (19.0)	5 (22.7)	
Secondary	24 (55.8)	15 (71.4)	9 (40.9)	
Tertiary	10 (23.3)	2 (9.5)	8 (36.4)	
**EMPLOYMENT** *n* (%)				0.566
Employed	20 (46.5)	8 (38.1)	12 (54.5)	
Unemployed	4 (9.3)	1 (4.8)	3 (13.6)	
Retired	5 (11.6)	2 (9.5)	3 (13.6)	
Sick leave	5 (11.6)	4 (19.0)	1 (4.5)	
Work-disabled	9 (20.9)	6 (28.6)	3 (13.6)	

SD: standard deviation. * Comparison between different groups. Repeated-measures one-factor ANOVA test.

**Table 2:** Mean patient scores for NRS, ODI, PGI, and CGI.

	NRS Mean ± SD				ODI Mean ± SD				PGI Mean ± SD				CGI Mean ± SD			
	Total (*n* = 43)	Stress (*n* = 21)	No Stress (*n* = 22)	*p*-Value *	Total (*n* = 43)	Stress (*n* = 21)	No Stress (*n* = 22)	*p*-Value *	Total (*n* = 43)	Stress (*n* = 21)	No Stress (*n* = 22)	*p*-Value *	Total (*n* = 43)	Stress (*n* = 21)	No Stress (*n* = 22)	*p*-Value *
**Baseline**	7.74 ± 1.17	7.61 ± 1.32	7.86 ± 1.03	0.502	43.79 ± 11.96	41.76 ± 13.69	45.72 ± 9.98	0.283	-	-	-	-	-	-	-	-
**1 Month**	5.51 ± 2.64	6.19 ± 2.74	4.86 ± 2.41	0.100	42.20 ± 12.00	44.61 ± 12.59	39.90 ± 11.22	0.202	3.11 ± 0.93	3.42 ± 0.87	2.81 ± 0.90	0.030	2.11 ± 0.90	2.42 ± 0.81	1.81 ± 0.90	0.025
**3 Months**	5.46 ± 2.67	6.00 ± 2.81	4.95 ± 2.49	0.204	41.58 ± 12.83	44.09 ± 13.45	39.18 ± 12.02	0.213	0.92 ± 1.06	3.23 ± 1.17	2.77 ± 0.92	0.156	2.04 ± 0.99	2.33 ± 1.01	1.77 ± 0.92	0.065
**6 Months**	5.53 ± 2.65	6.09 ± 2.79	5.00 ± 2.46	0.180	42.00 ± 12.88	45.33 ± 14.03	38.81 ± 11.08	0.098	3.04 ± 1.09	3.33 ± 1.19	2.77 ± 0.92	0.092	2.09 ± 1.01	2.42 ± 1.02	1.77 ± 0.92	0.033
**12 Months**	6.18 ± 2.61	7.23 ± 2.54	5.18 ± 2.30	0.008	42.27 ± 11.43	46.28 ± 11.05	38.45 ± 10.67	0.023	3.32 ± 1.04	3.71 ± 0.95	2.95 ± 0.99	0.015	2.69 ± 1.16	3.19 ± 1.03	2.22 ± 1.10	0.005
***p*** -value **	0.001	0.044			0.093	0.011			0.895	0.026			0.540	0.011		

SD: standard deviation; NRS: Numeric Rating Scale; ODI: Oswestry Disability Index; PGI: Patient Global Impression of Improvement; CGI: Clinical Global Impression of Improvement. * Comparison between scores for stress and no-stress groups at a specific time during follow-up. Repeated-measures one-factor ANOVA test. ** Comparison between scores throughout follow-up for (1) total and (2) stress and no stress groups together. Repeated-measures one-factor ANOVA test.

**Table 3:** Mean patient scores for SF12.

	SF Physical Mean ± SD				SF Mental Mean ± SD				SF Total Mean ± SD			
	Total (*n* = 43)	Stress (*n* = 21)	No Stress (*n* = 22)	*p*-Value *	Total (*n* = 43)	Stress (*n* = 21)	No Stress (*n* = 22)	*p*-Value *	Total (*n* = 43)	Stress (*n* = 21)	No Stress (*n* = 22)	*p*-Value *
**Baseline**	19.18 ± 16.96	24.20 ± 21.67	14.39 ± 8.88	0.057	29.80 ± 13.87	31.70 ± 15.37	27.99 ± 12.36	0.386	24.49 ± 14.06	27.95 ± 17.64	21.19 ± 8.70	0.116
**1 Month**	25.57 ± 11.26	23.80 ± 13.83	27.27 ± 8.10	0.319	41.62 ± 21.91	33.72 ± 14.42	49.16 ± 25.31	0.019	33.60 ± 14.78	28.77 ± 12.48	38.22 ± 15.59	0.034
**3 Months**	29.35 ± 20.79	27.57 ± 25.60	31.05 ± 15.30	0.589	46.19 ± 23.67	38.72 ± 20.89	53.33 ± 24.40	0.042	37.78 ± 20.11	33.15 ± 22.61	42.19 ± 16.74	0.143
**6 Months**	28.97 ± 20.97	28.17 ± 25.37	29.73 ± 16.28	0.811	46.58 ± 24.07	38.84 ± 20.59	53.97 ± 25.26	0.038	37.78 ± 20.36	33.51 ± 22.34	41.85 ± 17.85	0.183
**12 Months**	27.22 ± 18.68	26.78 ± 19.87	27.65 ± 17.93	0.023	45.91 ± 24.17	37.97 ± 18.68	53.48 ± 26.71	0.034	36.56 ± 17.96	32.38 ± 16.92	40.56 ± 18.39	0.137
***p*** -value **	0.002	0.088			0.001	0.030			0.001	0.026		

SD: standard deviation; SF12: Short Form 12 Health Survey. * Comparison between scores for stress and no stress groups at a specific time during follow-up. Repeated-measures one-factor ANOVA test. ** Comparison between scores throughout follow-up for (1) total and (2) stress and no stress groups together. Repeated-measures one-factor ANOVA test.

**Table 4:** Mean patient scores for MOS Sleep Scale subscales.

	SLPD4 Disturbances Mean ± SD				SLPNR1 Snoring Mean ± SD				SLPSOB1 Shortness of Breath Mean ± SD				SLPA2 Adequacy Mean ± SD			
	Total (*n* = 43)	Stress (*n* = 21)	No Stress (*n* = 22)	*p*-Value *	Total (*n* = 43)	Stress (*n* = 21)	No Stress (*n* = 22)	*p*-Value *	Total (*n* = 43)	Stress (*n* = 21)	No Stress (*n* = 22)	*p*-Value *	Total (*n* = 43)	Stress (*n* = 21)	No Stress (*n* = 22)	*p*-Value *
**Baseline**	60.72 ± 22.48	56.13 ± 18.65	65.11 ± 25.26	0.194	47.90 ± 27.99	43.80 ± 29.40	51.81 ± 26.66	0.355	24.65 ± 16.81	22.85 ± 15.85	26.36 ± 17.87	0.501	30.46 ± 17.31	33.33 ± 18.52	27.72 ± 16.01	0.294
**1 Month**	54.82 ± 15.64	54.82 ± 12.10	54.82 ± 18.70	0.999	46.97 ± 26.14	40.95 ± 25.67	52.72 ± 25.85	0.142	24.18 ± 19.30	24.76 ± 15.36	23.63 ± 22.79	0.851	36.51 ± 21.91	38.57 ± 22.42	34.54 ± 21.76	0.553
**3 Months**	55.17 ± 14.67	54.70 ± 11.42	55.62 ± 17.48	0.840	43.72 ± 24.78	36.19 ± 22.46	50.90 ± 25.24	0.050	24.18 ± 19.30	24.76 ± 15.36	23.63 ± 22.79	0.851	35.58 ± 20.96	39.04 ± 2.64	32.27 ± 17.97	0.295
**6 Months**	55.17 ± 14.67	54.70 ± 11.42	55.62 ± 17.48	0.840	43.72 ± 24.78	36.19 ± 22.46	50.90 ± 25.24	0.050	24.18 ± 19.30	24.76 ± 15.36	23.63 ± 22.79	0.851	34.88 ± 20.97	39.04 ± 23.64	30.90 ± 17.70	0.207
**12 Months**	51.71 ± 15.06	50.35 ± 12.77	53.01 ± 17.16	0.570	41.44 ± 25.29	34.28 ± 23.78	48.27 ± 25.35	0.069	19.06 ± 15.70	18.09 ± 10.77	20.00 ± 19.51	0.696	34.65 ± 22.18	38.57 ± 24.14	30.90 ± 19.97	0.263
***p*** -value **	0.006	0.539			0.026	0.482			0.001	0.538			0.218	0.830		
	**SLPS3 Somnolence** **Mean ± SD**					**SLP6 Problems Index I** **Mean ± SD**					**SLP9 Problems Index II** **Mean ± SD**					
	**Total** **(*n* = 43)**		**Stress** **(*n* = 21)**	**No Stress** **(*n* = 22)**	***p*** **-** Value *	**Total** **(*n* = 43)**		**Stress** **(*n* = 21)**	**No Stress** **(*n* = 22)**	***p*** **-** Value *	**Total** **(*n* = 43)**		**Stress** **(*n* = 21)**	**No Stress** **(*n* = 22)**	***p*** **-** Value *	
**Baseline**	37.67 ± 11.44		38.41 ± 13.80	36.96 ± 8.90	0.685	50.85 ± 12.74		48.41 ± 12.09	53.18 ± 13.19	0.224	52.51 ± 14.03		49.92 ± 13.03	54.99 ± 14.79	0.240	
**1 Month**	37.73 ± 12.22		32.56 ± 12.22	37.27 ± 11.93	0.165	44.26 ± 12.43		44.44 ± 11.51	44.09 ± 13.52	0.927	47.57 ± 13.21		47.01 ± 11.60	48.10 ± 14.85	0.790	
**3 Months**	34.26 ± 30.61		30.16 ± 15.72	38.18 ± 10.11	0.052	44.41 ± 11.92		43.65 ± 12.60	45.15 ± 11.48	0.685	47.93 ± 12.27		46.53 ± 12.01	49.26 ± 12.65	0.472	
**6 Months**	34.26 ± 13.61		30.16 ± 15.72	38.18 ± 10.11	0.052	44.57 ± 11.05		43.65 ± 12.60	43.65 ± 12.60	0.620	47.98 ± 12.19		46.53 ± 12.01	49.37 ± 12.49	0.453	
**12 Months**	39.06 ± 11.78		38.41 ± 13.64	39.69 ± 9.96	0.725	43.79 ± 9.61		43.17 ± 9.03	44.39 ± 10.30	0.683	46.44 ± 10.97		45.02 ± 9.79	47.80 ± 12.07	0.414	
***p*** -value **	0.557		0.279			0.001		0.264			0.007		0.637			

SD: standard deviation. * Comparison between scores for stress and no stress groups at a specific time during follow-up. Repeated-measures one-factor ANOVA test. ** Comparison between scores throughout follow-up for (1) total and (2) stress and no stress groups together. Repeated-measures one-factor ANOVA test.

**Table 5:** Change at 12 months post-intervention for all studied outcome measures.

	Total N = 43 (100)		Stress N = 21 (48.8)		No Stress N = 22 (51.2)	
	Mean	Patients over MCID (%)	Mean	Patients over MCID (%)	Mean	Patients over MCID (%)
**NRS** (MCID ≥ 2)	−1.56	86.0	−0.38	81.0	−2.68	90.9
**ODI** (MCID ≥ 10)	−1.51	14.0	4.52	23.8	−7.27	4.5
**PGI** (MCID ≥ 1)	3.33	100	3.71	100	2.95	100
**CGI** (MCID ≥ 1)	2.70	100	3.19	100	2.22	100
**SF12 Physical** (MCID ≥ 3.29)	8.04	14.0	2.58	14.3	13.26	13.6
**SF12 Mental** (MCID ≥ 3.77)	16.10	55.8	6.27	33.3	25.49	77.3
**SF12 Total** (MCID ≥ 7.06)	12.07	55.8	4.43	38.1	19.37	72.7
**SLPD4** (MCID ≥ 6)	−9.01	25.6	−5.78	28.6	−12.10	22.7
**SLPSNR1** (MCID ≥ 6)	−6.47	2.3	−9.52	4.8	−3.54	0
**SLPSOB1** (MCID ≥ 6)	−5.58	0	−4.76	0	−6.36	0
**SLPA2** (MCID ≥ 6)	4.19	11.6	5.24	19.0	3.18	4.5
**SLPS3** (MCID ≥ 6)	1.40	41.9	0.00	33.3	2.73	50.0
**SLP6** (MCID ≥ 6)	−7.05	2.3	−5.24	4.8	−8.79	0
**SLP9** (MCID ≥ 6)	−6.07	16.3	−4.90	23.8	−7.19	9.1

MCID: minimally clinically important difference.

## Data Availability

The authors certify that this manuscript reports original data that will not be made publicly available.
